# A POSSIBLE COMPLICATION AFTER LIVER TRANSPLANTATION IN A GASTRIC BYPASS BARIATRIC PATIENT: DON’T FORGET THE EXCLUDED STOMACH! CASE REPORT AND REVIEW OF THE LITERATURE

**DOI:** 10.1590/0102-672020190001e1510

**Published:** 2020-08-24

**Authors:** Pietro PERDUCA, Daniel Reis WAISBERG, Rafael Soares Nunes PINHEIRO, Eduardo Guimarães HOURNEAUX-DE-MOURA, Luiz Augusto Carneiro D’ALBUQUERQUE, Wellington ANDRAUS

**Affiliations:** 1 Grand Hôpital de Charleroi - Saint-Joseph, Service of Digestive Surgery, Gilly, Belgium; 2 Hospital das Clínicas, Faculty of Medicine, University of São Paulo, Department of Gastroenterology, São Paulo, SP, Brazil

**Keywords:** Organ transplantation, Obesity, Bariatric surgery, Peptic ulcer perforation, Gastric stump, Transplante de órgãos, Obesidade, Cirurgia bariátrica, Úlcera péptica perfurada, Coto gástrico

## INTRODUCTION

Obesity is a global epidemic^8^. Surgery has proven to be the most effective treatment for morbid obesity^8^. The estimated prevalence of non-alcoholic fatty liver disease in obese is three times higher than in the general population^8^. It progresses to non-alcoholic steatohepatitis in up to 42% of cases, which has become a growing indication for liver transplantation (LT)^8^. Bariatric surgery in patients with cirrhosis prior to LT may improve access in the waiting list. The number of patients on the waiting list for transplantation having undergone bariatric surgery will grow, with a potential increase in the rate complications. Peptic ulcer (PU) perforation is one of them. Following Roux-en-Y gastric bypass (RYGBP), the modified anatomy and physiology are a risk factor for peptic ulceration of an excluded stomach. Furthermore, LT carries specific risk factors for PU. Diagnosis in the gastric remnant can be challenging due to the absence of endoscopic access. 

We report the case of a LT recipient suffering from a perforated PU in the bypassed stomach from RYGBP. To our knowledge, this is the first case reported in a liver transplanted patient.

## CASE REPORT

A 45-year-old woman with a history of open Fobi-Capella RYGBP was diagnosed with primary biliary cirrhosis and listed for LT. Bariatric surgery was carried out seven years before, followed by an emergency reintervention for obstruction of the jejunojejunostomy. Hepatopathy was diagnosed at 41 years of age. The patient presented Ig G antibodies for cytomegalovirus and a negative viral DNA detection by quantitative PCR. There were no other relevant comorbidities.

She was admitted to the emergency department with melena and hematochezia. Physical examination revealed hypotension, paleness, icterus and a pain-free abdomen without ascites. Her Model for End-Stage Liver Disease score was 33. The patient did not smoke, consume alcohol to excess or use nonsteroidal anti-inflammatory drugs, acetylsalicylic acid, or proton pump inhibitors. The *Helicobacter pylori* (HP) status was unknown, nor it was investigated. The patient was clinically managed with intravenous crystalloids, blood borne products transfusion, PPI and ciprofloxacin. The upper endoscopy was negative and the abdominal Doppler ultrasound showed signs of portal hypertension with patent hepatic vessels. Six days after admission, deceased donor LT was carried out without perioperative complications.

**FIGURE 1:**
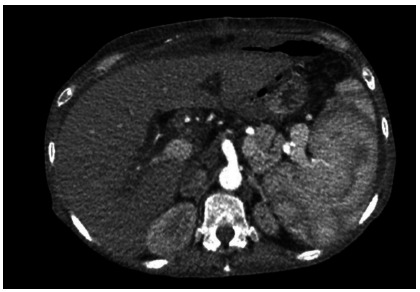
Abdominal computed tomography scan with intravenous contrast at arterial phase showing free air anterior to the stomach, periportal edema of the transplanted liver and splenomegaly.

The postoperative immunosuppression regimen consisted of prednisone, tacrolimus and mycophenolate sodium. The prophylactic antibiotics consisted of amikacin and ampicillin until postoperative day (POD) 2 and ivermectin on PODs 2 and 3; sulfamethoxazole was introduced on POD 8. Acetylsalicylic acid and prophylactic low molecular weight heparin were suspended from POD 3 to POD 7 because of anemization without signs of bleeding. Low molecular weight heparin was reintroduced at therapeutic dose because of the thrombosis of a branch of the right portal vein. On POD 7 hepatic biopsy was performed due to elevation in liver enzymes. Moderate acute cellular rejection was diagnosed and treated with pulse therapy of methylprednisolone. Proton pump inhibitors were administered throughout the hospitalization. On POD 14 the patient developed an acute abdomen. An abdominal computed tomography scan with intravenous contrast showed a pneumoperitoneum with foci of free air next to the stomach and free abdominal fluid in small quantity (Figure 1). 

An emergency laparotomy was performed and a perforated ulcer of the body of the excluded stomach was found and repaired by simple closure. The ulcer was not resected for pathological examination. On POD 16 routine quantitative PCR for cytomegalovirus DNA was positive (41UI/ml 1,62 log (UI/ml)), but did not require antiviral therapy nor reduction in the immunosuppressive regimen. Prophylactic unfractioned heparin was administered from POD 16. Culture of the abdominal liquid collected intraoperatively showed positive for extended spectrum beta-lactamase producing *Klebsiella pneumoniae* and *Enterococus faecium*. Antibiotic treatment consisted of vancomycin, meropenem and fluconazol. The patient was discharged on POD 26 with immunosuppressors, sulfamethoxazole, proton pump inhibitors and prophylactic low molecular weight heparin, the latter being discontinued ten days after this.

During follow-up, PCR for cytomegalovirus DNA showed negative results six weeks after discharge. Proton pump inhibitors were continued on double dose. Seven months after discharge, the patient underwent a double-balloon enteroscopy exploration of the excluded stomach - the second reported after a PU perforation in the gastric remnant^13^. Because of the enteroenterostomies created after the RYGBP, it was impossible to reach the excluded stomach. Pathological examination of biopsies from the gastric pouch and the alimentary limb was negative for inflammatory alterations, cytomegalovirus or HP. Three years after transplantation, the patient remains well undergoing routine outpatient evaluation. 

## DISCUSSION

Reports of PU perforation in the excluded stomach after RYGBP are rare. A Pubmed search limited to articles in English found only 29 reported cases (Table 1). The incidence of perforated PU after open RYGBP in the Macgregor et al. series is 0.25%^10^. Based on the review, the male to female ratio is 1:1.9. The age at the time of PU perforation ranges from 24 to 63 years (mean 42.6). The delay between the RYGBP and the presentation of the perforated PU ranges from five days to 13 years. Twenty-one patients had duodenal ulcer perforation (72.4%), seven had gastric ulcer perforation (24.1%), and one had both (3.4%, Table 1). Statistics on PU after LT were not found in the literature.

**TABLE 1:** Summary of all reported cases of excluded stomach perforation

author, year of publication	number of cases	sex	age	delay RYGBP - PU perforation	site of perforation	pneumoperitoneum (imaging tool)	emergency treatment (technique)	emergency surgical procedure	definitive treatment
Moore et al., 1979^12^	2	m	28	12 d	duodenum	NA	surgical (laparotomy)	omentopexy	-
		m	53	5 d	duodenum	NA	surgical	NA	-
Andersen et al., 1982^1^	1	f	34	3 y	stomach	no (XR)	medical	-	closure + RYGBP takedown
Charuzi et al., 1986^4^	2	NA	NA	3 w	duodenum	no (opacification through gastrostomy)	surgical	omentopexy	-
		NA	NA	6 mo	duodenum	yes (opacification through gastrostomy)	surgical	omentopexy	-
Bjorkman et al., 1989^3^	1	m	24	6 y	duodenum	no (US)	surgical	closure + gastrectomy	-
Macgregor et al., 1999^10^	11	f	63	23 mo	duodenum	no	surgical	closure	gastrectomy
		f	37	21 mo	stomach	no	surgical	closure + gastrostomy	gastrectomy
		f	40	8 y	duodenum	no	surgical	closure	medical
		f	31	7 mo	duodenum	no	surgical	closure + gastrostomy + cholecystectomy	gastrectomy
		f	53	5 y	duodenum	no	surgical	closure + vagotomy + pyloroplasty	-
		f	43	8 y	duodenum	no	surgical	closure	gastrectomy
		f	29	11 y, 4 mo	duodenum	no	surgical	closure	gastrectomy
		m	48	4 y	duodenum	no	surgical	closure	gastrectomy
		f	57	18 mo	duodenum and stomach	no	surgical	closure	gastrectomy
		m	40	20 d	duodenum	no	surgical	closure	gastrectomy
		f	56	12 y	duodenum	no	surgical	unsuccessful closure then drainage + gastrostomy	gastrectomy
Papasavas et al., 2003^14^	1	f	35	1 y	stomach	yes (XR)	surgical (laparotomy)	partial gastrectomy	medical
Arshava et al., 2006^2^	1	m	36	3 y	stomach	no (XR, CT)	surgical (laparotomy)	gastrectomy + cholecystectomy	-
Mittermair and Renz, 2007^11^	1	f	54	15 mo	duodenum	yes (CT)	surgical (laparoscopy)	closure + omentopexy	-
Snyder, 2007^17^	4	NA	NA	NA	3 duodenum, 1 stomach	NA	surgical	1 closure, 3 gastrectomies	-
Sasse et al., 2008^16^	1	f	55	1 y	stomach	yes (XR)	surgical	closure + omentopexy	-
Gypen et al., 2008^5^	1	f	35	10 w	duodenum	no (XR, US)	surgical (laparoscopy)	closure + omentopexy + cholecystectomy	medical
Iskandar et al., 2015^6^	2	m	59	10 y	duodenum	yes (CT)	surgical (laparoscopy)	closure + omentopexy	-
		m	37	13 y	duodenum	no (CT)	surgical (laparotomy)	drainage + jejunostomy	medical
Ovaere et al., 2016^13^	1	f	33	14 m	stomach	no (US, CT)	surgical (laparoscopy)	closure + omentopexy	medical

CT=abdominal computed tomography scan; d=days; f=female; gastrectomy=gastrectomy of the bypassed stomach; m=male; mo=months; NA=not available; PU=peptic ulcer; RYGBP=Roux-en-Y gastric bypass; RYGBP takedown=gastrogastrostomy between proximal and distal gastric pouches, removal of the Roux-en-Y and reconstruction with a jejunojejunostomy; US=abdominal ultrasound; w=weeks; XR=plain abdominal X ray film (except in Papasavas et al.^14^, which used chest X ray film); y=years.

The anatomical and physiological modifications after RYGBP may contribute to PU pathogenesis in the bypassed stomach. Acid production may be promoted by hormonal and vagal stimuli, which cannot be buffered by the ingested food or by a physiological pancreatic secretion of bicarbonate, and by small gastric pouches, increasing the parietal cell mass of the distal remnant^3^ ^,^ ^10^. Excluded gastric mucosa is also exposed to chronic injury and possibly carcinogenesis by duodenogastric bile reflux, as demonstrated by double-balloon enteroscopy^15^. This technique detected HP in 20% of the excluded stomachs and the severity of gastritis was associated with positive HP status^15^. As all HP positive patients in the excluded stomach were also positive in the gastric pouch, HP detection in the excluded stomach may therefore be unnecessary^15^. PU disease in the excluded stomach shares the same risk factors of general PU or marginal ulcers, but solid organ transplantation also has distinctive risk factors, especially regarding immunosuppressive therapy. After kidney transplantation, high-dose corticosteroids for rejection are associated with a greater rate of gastric ulceration^9^. Moreover, mycophenolate mofetil slows the gastric cell regeneration cycle^9^. Among infections, cytomegalovirus is the most common pathogen complicating solid organ transplantation^7^.

The diagnosis of a perforated PU in the gastric remnant can be delayed. Pneumoperitoneum in imaging is rare probably because the air in the excluded stomach is progressively absorbed. Computed tomography is the most accurate diagnostic exam. Double-balloon enteroscopy could be useful in gastrointestinal bleeding of unknown origin following RYGBP, as bleeding preceded perforation in our case and in two other reported cases^3^ ^,^ ^14^. In case of gastrointestinal complications after LT, cytomegalovirus invasive disease should be ruled out by combining tests for active disease, such as quantitative PCR, with immunohistochemistry on biopsies to maximize sensitivity^7^. The differential diagnosis includes secondary perforation for an internal hernia or gastric malignancy.

Several surgical or endoscopic treatments are available for general PU perforation. Sepsis is the priority of postoperative care^18^. Administration of early broad-spectrum intravenous antibiotics is important, though the effect of antifungal therapy is not clear^18^. Moreover, HP eradication reduces the incidence of PU recurrence^18^. In the setting of a perforated PU in the defunctionalized stomach, the most commonly reported emergency treatment is surgical and consists in a simple closure (Table 1). Data on postoperative proton pump inhibitors, antibiotics, HP eradication and prophylactic anticoagulation are poor. Some authors propose gastrectomy of the gastric remnant as the definitive treatment in case of perforation, others suggest primary resection concurrent to the RYGBP^5^ ^,^ ^10^ ^,^ ^17^. Arguments in support of gastrectomy of the bypassed stomach include the exclusion of the follow-up of the nearly inaccessible gastric remnant, the absence of gastrogastric fistulas and the possible reduction of stomal ulcers by resecting the gastrin-releasing part of the stomach^6^. However the disadvantages may be the bleeding of omental vessels, omental fat necrosis with abscess formation, duodenal stump leakage, prolongation of operative time, bacterial overgrowth in the biliopancreatic limb and vitamin B12 deficiency^5^ ^,^ ^6^. Therefore, long term proton pump inhibitors therapy could be an alternative for high risk patients^6^. Gastrectomy of the excluded stomach during LT has never been reported. Finally, prevention and treatment of cytomegalovirus infection must be rigorous in patients with RYGBP to avoid, among other complications, gastrointestinal perforation.
